# Primary care physicians' perspectives and challenges on managing multimorbidity for patients with dementia: a Japan–Michigan qualitative comparative study

**DOI:** 10.1186/s12875-023-02088-4

**Published:** 2023-06-27

**Authors:** Shinji Tsunawaki, Michiko Abe, Melissa DeJonckheere, Christine T. Cigolle, Kristin K. Philips, Ellen B. Rubinstein, Masakazu Matsuda, Michael D. Fetters, Machiko Inoue

**Affiliations:** 1 Omaezaki Family Medicine Center, Omaezaki, Shizuoka Japan; 2 Shizuoka Family Medicine Program, Shizuoka, Hamamatsu Japan; 3 Department of Family and Community Medicine, Hamamatsu University School of Medicine, Hamamatsu, Shizuoka Japan; 4 Department of Family Medicine, University of Michigan, Ann Arbor, MI USA; 5 Department of Internal Medicine, University of Michigan, Ann Arbor, MI USA; 6 Education and Clinical Center (GRECC), VA Ann Arbor Healthcare System (VAAAHS) Geriatric Research, Ann Arbor, MI USA; 7 Department of Sociology and Anthropology, North Dakota State University, Fargo, ND USA; 8 Kikugawa Family Medicine Center, Kikugawa, Shizuoka Japan; 9 The School of Health Humanities, Peking University Health Science Center, Beijing, China

**Keywords:** Multimorbidity management, Primary care physicians, Japan-US comparison, Qualitative study, Dementia, Multiple chronic conditions, Guidelines, Care resources, Multidisciplinary support

## Abstract

**Background:**

Multimorbidity management can be extremely challenging in patients with dementia. This study aimed to elucidate the approaches of primary care physicians in Japan and the United States (US) in managing multimorbidity for patients with dementia and discuss the challenges involved.

**Methods:**

This qualitative study was conducted through one-on-one semi-structured interviews among primary care physicians, 24 each from Japan and Michigan, US. Thematic and content analyses were performed to explore similarities and differences among each country’s data.

**Results:**

Primary care physicians in Japan and Michigan applied a relaxed adherence to the guidelines for patients’ chronic conditions. Common challenges were the suboptimal consultation time, the insufficient number or ability of care-coordinating professionals, patients’ conditions such as difficulties with self-management, living alone, behavioral issues, and refusal of care support. Unique challenges in Japan were free-access medical systems and not being sure about the patients’ will in end-of-life care. In Michigan, physicians faced challenges in distance and lack of transportation between clinics and patients’ homes and in cases where patients lacked the financial ability to acquire good care.

**Conclusions:**

To improve the quality of care for patients with multimorbidity and dementia, physicians would benefit from optimal time and compensation allocated for this patient group, guidelines for chronic conditions to include information regarding changing priority for older adults with dementia, and the close collaboration of medical and social care and community resources with support of skilled care-coordinating professionals.

**Supplementary Information:**

The online version contains supplementary material available at 10.1186/s12875-023-02088-4.

## Background

Multimorbidity is defined as the coexistence of two or more long-term conditions [1–3]. If dementia is one of the multiple chronic conditions of a patient, multimorbidity management can become more challenging [4–7]. In recent years, several guidelines for multimorbidity management have been published based on experts’ opinions [3, 8–11]. Their recommendations emphasize providing individualized care by incorporating patients’ preferences [3, 8, 9, 11, 12], accurately interpreting evidence [8], and considering the benefits and risks by following the guidelines for each separate condition [3] and taking into account the clinical feasibility [8] or setting realistic treatment goals [12]. However, dementia is simply one of the chronic conditions in those recommendations. The difficulties of managing multiple diseases in patients with cognitive decline have not been adequately discussed.

Previous studies have reported that people with dementia are less likely to receive the same quality of care or access to services as those without dementia [6]. For example, individuals with dementia may be prescribed inappropriate medications as chronic conditions increase [13]. They may also be less likely to receive hemoglobin A1c tests, low density lipoprotein cholesterol (LDL-C) tests, or eye examinations for diabetes mellitus monitoring [14], and have poorer cancer-related outcomes, including a later stage cancer diagnosis [15]. Those studies focused on the management of each specific disease, but it is also worth considering how primary care physicians provide care to the patients with various chronic conditions as a whole person, in order to improve their quality of life, while avoiding unnecessary treatment. Individuals with dementia may have difficulty organizing care, such as keeping clinical visits or taking medications, and cannot report symptoms to their family or healthcare professionals [4, 16]. For healthcare providers, barriers in managing this patients group include a lack of data on the efficacy and safety of most medications for people with dementia [17], communication complexities that involve the patient’s family and multidisciplinary professionals [18–20], and lack of resources for collaboration, such as healthcare resources, pharmacists, and specialists [8, 11]. These patients require comprehensive and personalized care, and for that reason, the care practice is affected by the local healthcare system and care resources [11, 21]. Healthcare providers in each location may have unique challenges or ways to negotiate with situations.

Japan has the most aging population in the world, and multimorbidity management for older patients is a major concern in primary care [22, 23]. On the other hand, Japanese medicine and healthcare has been managed by organ-based specialties, and the education for general practice or family medicine is still in the emerging phase [24, 25]. It remains unknown how Japanese primary care physicians approach patients with complex needs such as multimorbidity with dementia. The University of Michigan is a major contributor to training Japanese family physicians through exchanges of medical students and educators [24, 26]. By comparing the experiences of primary care physicians in Japan to those of the counterparts in Michigan where family practice is more situated, we may be able to understand the universal challenges in managing multimorbidity in patients with dementia, as well as to explore how different cultures and contexts influence the experience of caring for such patients. The research questions of the present study are: (1) What are the approaches of primary care physicians in Japan and Michigan in the United States (US) for managing multimorbidity for patients with dementia, (2) What are the common and unique challenges they face in such practice? Based on the results, we sought to discuss the resources or supports needed for primary care physicians to provide quality multimorbidity care for patients with dementia.

## Methods

**Fig. 1:**
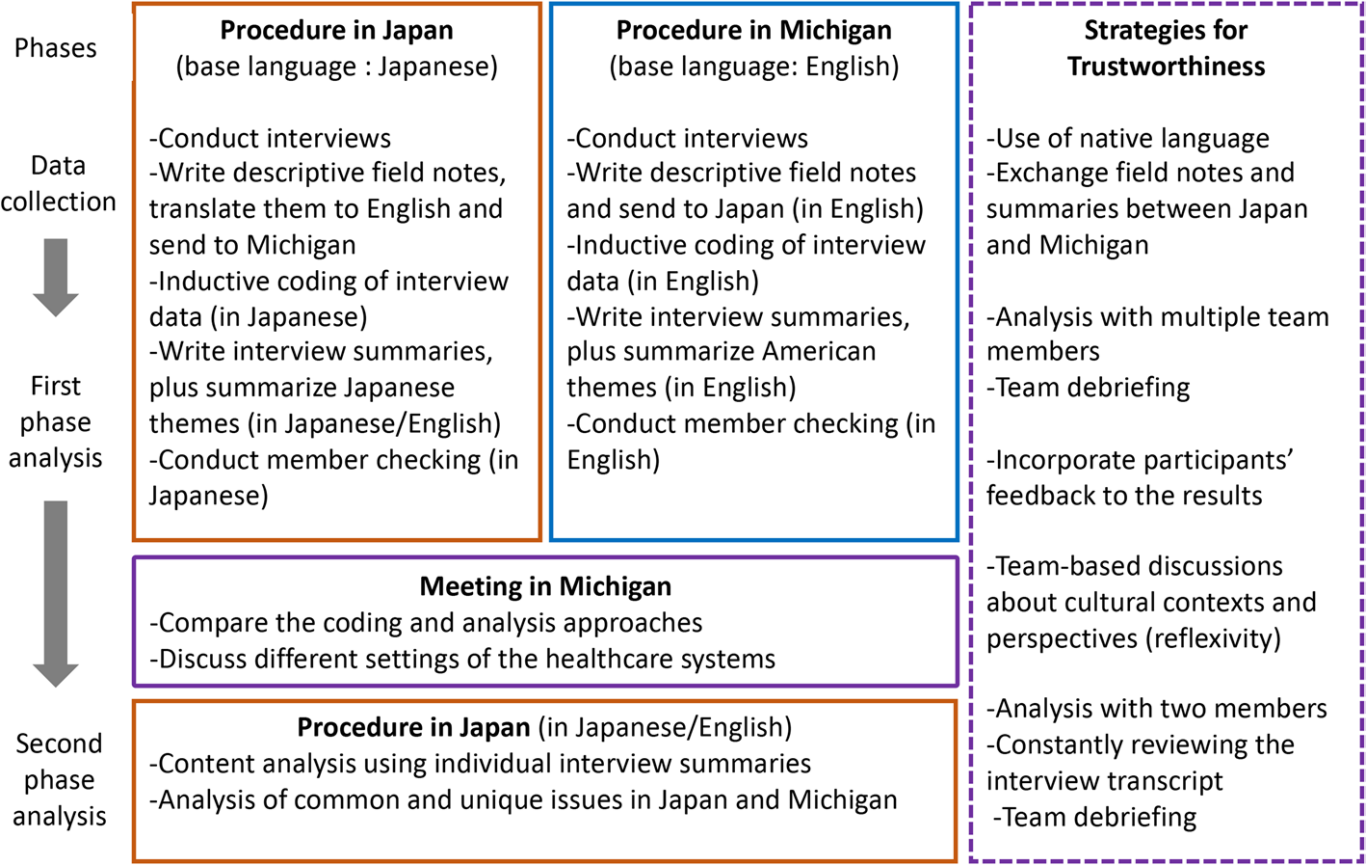
Research procedure and strategies to enhance trustworthiness

This study was organized through one-on-one semi-structured interviews with primary care physicians. We used a qualitative descriptive design based on the pragmatic worldview to focus on the research problem and to apply useful approaches for understanding the phenomena of our interest [27, 28]. The qualitative description produces a focused summary and understanding of health-related experiences that includes contextual cultural factors that shape the participants’ experiences [29]. This approach was necessary for our study owing to the lack of research on primary care physicians’ management of multimorbidity including dementia. Two approaches were undertaken based on the research questions. In the first phase, inductive coding of interview transcripts from each country was used to discover patterns and develop themes that independently emerged from each country’s data [27] regarding the management of multimorbid patients with dementia. In the second phase, content analysis by deductive coding [30, 31], based on each country’s interview summaries and transcripts, was used to understand the different levels of practice challenges. Finally, the analysis from both countries were compared. As a summary of the study methods, Fig. 1 shows the procedure of data collection and interpretation in conducting the comparative study in Japan and Michigan. Details of the data collection, analysis, and strategies for trustworthiness are described in detail below. This study is a part of a larger investigation comparing rural and urban primary care physicians’ dementia care for multimorbid older adults in Japan and the US. The methodological details of the larger study are described elsewhere [32].

### Setting

The present study considers some characteristics of the healthcare systems in Japan and the US regarding primary care [32]. First, family practice in the US has been recognized as a specialty since the 1960s and they are systematically trained to provide care for patients of various ages in a continuous relationship [33]. Patients usually require a referral from their primary care doctors to see a specialist, as an approach to help minimize health care costs [34]. In Japan, family practice or general practice is a relatively new field, with a gradual growth in 2000-2010s, and many primary care physicians in Japan practice with subspecialties [35, 36]. Unlike in the US, patients in Japan have free access to specialists and can visit multiple physicians without a referral.

Second, Japan has universal health coverage and a long-term care insurance system [37, 38]. To respond to the rapid growth of the aging population, the government has been promoting a community-based integrated care system since 2012 and activating seamless healthcare services with a multidisciplinary approach [39, 40]. On the other hand, private health insurance is the predominant source of health insurance coverage in the US. For the citizens aged 65 and older, there have been two primary types of public health insurance systems: Medicare and Medicaid for low-income citizens and others meeting certain disease criteria [41, 42]. Medical and long-term care insurance in the US is largely under the control of the individual depending on their employment history and financial strength, and unlike Japan’s universal access, long-term care insurance in the US is very expensive and available through private insurers [43].

### Data collection instrument

The initial interview guide was developed in English by CC (family physician and geriatrician), MF (family physician and research methodologist), and a medical and public health student based on a review of the literature and clinical experience. The medical student piloted the interview guide with peers to assess its flow and wording. After the initial enrollment of two participants, we further refined the instrument iteratively primarily to address its flow and encourage more elaboration. For use in Japan, after translation to Japanese by a professional service, two bilingual senior investigators (MI and MF) reviewed the content and confirmed the language and substance of the inquiry to be natural and appropriate for the Japanese context.

Interview questions addressed primary care physicians’ goals in managing multimorbid patients with dementia, their practices of diagnosing, disclosing, and managing dementia; their comfort level with these tasks; and available resources for dementia care in their working environment (Supplement 1). We asked each participant to describe memorable patient cases and their trajectories, and we probed their perspectives and approaches to managing patients whose multimorbidity included dementia. We used a semi-structured interview format that allowed the interviewer to ask follow-up questions to probe for further information on topics of interest [44]. Although the dementia care process was discussed broadly during the interview, this paper focuses on primary care physicians' practices in managing multimorbidity and dementia.

### Recruitment, sampling, and data collection procedures

Data collection was performed in Japan from August 2017 to October 2017 and in the US between August 2015 and June 2018. We aimed to recruit physicians practicing in rural and urban areas in each country by using our own network of professionals. At the time of recruitment, we explained by e-mail that the purpose of this interview was to learn about primary care physicians’ experiences and perspectives in managing older patients with dementia. In Japan, participants were recruited from physicians residing throughout the country who were affiliated with the Japan Primary Care Association. In the US, participants were recruited from primary care physicians in the state of Michigan, which has both rural and urban areas. In addition to the practice locations, we aimed to have a mixture of physicians based on sex, age, and the number of years of experience as a primary care physician in both countries in accordance with maximum variation sampling.

Four team members were assigned as interviewers: a qualitative research methodologist with training in intercultural communication (MA) in Japan, a geriatric pharmacist (KP), a medical anthropologist (ER), and a medical and public health student in the US. All interviewers were trained to conduct qualitative interviews and had no previous relationship with the participants.

Interviews were conducted either in person at the physicians’ offices, on the telephone/by voice over the internet, or by video conferencing. No one else was present besides the participants and the interviewer. Participants’ native language was used for interview that lasted 60 to 150 min in Japan and 30 to 90 min in the US. All were audio-recorded and transcribed verbatim. Repeat interviews were not carried out.

### Ethical consideration

Participants gave written consent indicating their acceptance of the voluntary nature of study participation and the freedom to withdraw from the study. Ethical approval for this human study was obtained from The Institutional Review Board of Hamamatsu University School of Medicine in Japan (No. 16–233) and Health Sciences and Behavioral Sciences Institutional Review Board at the University of Michigan in the US (IRB 00000246).

### Qualitative data analysis

After each interview, the interviewers wrote descriptive field notes to capture the interview context and the major issues discussed. The Japan team members translated their descriptive field notes into English to exchange the results with the Michigan team several times throughout the data collection.

In the first phase, each team proceeded independently with thematic analysis via inductive coding [45]. We performed inductive coding to the transcribed interview data in each language, English for Michigan data and Japanese for Japan data, which facilitated an understanding of physicians’ perspectives in their own words. Three authors (CC, KP, and MD) coded the Michigan data and ensured agreement in discussion with MF and ER. One author (MA) coded the Japan data and ensured agreement in discussion with ST, MM, and MI. MAXQDA Analytic Pro 12 was used for data organization and management. After conducting 24 interviews in each country, the research team members agreed that thematic saturation had been reached as there were no new codes generated from either set of interview transcripts. Member checking was performed by sending each participant a summary of their interview along with an overall summary of findings from both Michigan and Japan. In Japan, all 24 participants agreed with the distributed summary, and 22 participants provided additional comments. In the US, 24 participants were emailed information about the study findings, although one email did not function. The nine responding participants all indicated agreement with a few clarifications. The participants’ feedback was incorporated into the findings.

For Japan and Michigan comparison for the first phase, the lead analyst/the second author from Japan (MA) visited Michigan to compare the data coding and analysis approach with the Michigan team members (MF, MD, CC, KP). This ensured that the coding schemes equivalently functioned to examine constructs of mutual interest.

The second phase of analysis included content analysis [30] using the multilevel framework as described by Firlie & Shortell (2001) [31] to examine the participants’ challenges in each setting. First, ST and MA used the individual interview summary to categorize the content into the participants’ practice perspectives and their challenges into four levels: environment, system, team, and individual. Subsequently, we considered each content for Japan and Michigan to analyze the common and unique issues in each location. The summary description was reviewed by going back and forth with the interview transcripts to confirm the meaning of the participants’ words. Finally, all authors debriefed the findings.

### Reflexivity

A strength of this study is the multidisciplinary nature of the research team who participated in its design, implementation, and analysis [46]. While the interviewees were all physicians, the team included members whose backgrounds are purely in the social sciences – intercultural communication, educational psychology, and medical anthropology, specifically – not the biomedical sciences. While medical personnel are trained in a predominantly positivist approach to health care research, meaning they understand there to be an “objective” reality that can be captured and measured, social scientists are trained to question objectivity through interpretivism [47]. Thus, throughout the research process, biomedical scientists and social scientists discussed and debated the significance of certain pieces of data, drawing on their respective positions as insiders (i.e., fellow medical professionals) and outsiders (i.e., social scientists without medical training) to critique assumptions and refine their findings [48]. Team members also included a mix of Japanese and American researchers, which added another contextual layer of interpretation, as culturally specific practices could be identified and examined through team-based cross-cultural comparison [46].

## Results

A total of 48 primary care physicians, 24 each from Japan and Michigan, participated in the interviews. Their profiles are illustrated in Table 1. No participants were under the age of 16. The average years of practice experience was slightly longer for the Japanese participants (13 vs. 11.5). The gender ratio was 71% male in Japan vs. 42% male in Michigan. In Japan, physicians based in local hospitals, many of which had outpatient departments, were included because they actively provide primary care services. All the physicians from Michigan (100%) practiced in clinics. Because Japan is much more densely populated than Michigan, we used the participants’ own categorization of their practice setting (urban or rural).

**Table 1:** Characteristics of participants (*n* = 48)

	Japan	Michigan
	(*n* = 24)	(*n* = 24)
Years practicing as physician^a^	13 (10–19)	11.5 (6–21)
Gender^b^		
Male	17 (71%)	10 (42%)
Female	7 (29%)	14 (58%)
Clinical setting^b^		
Clinics	18 (75%)	24 (100%)
Hospital	6 (25%)	none
Regional setting^b^		
Rural	12 (50%)	12 (50%)
Urban	12 (50%)	12 (50%)

^a^Median (interquartile range)

^b^numbers (%)

### Multimorbidity management for patients with dementia – Japan–Michigan comparison

**Fig. 2:**
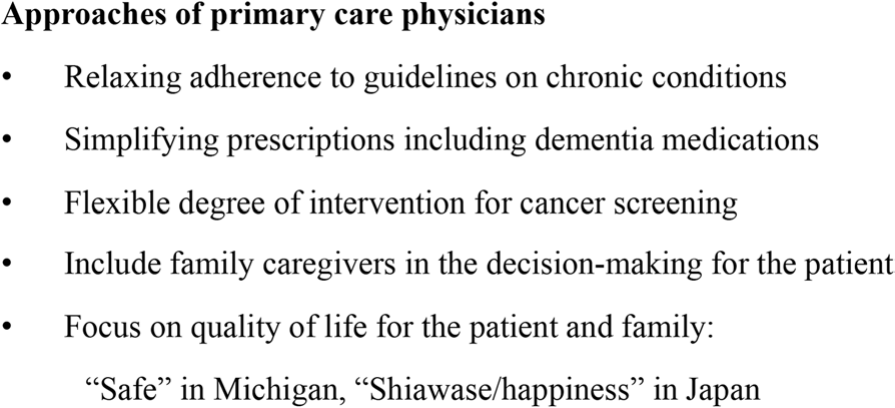
Approaches for managing multimorbidity in patients with dementia

The perspectives and practices of primary care physicians in Japan and Michigan were similar regarding the multimorbidity management of patients with dementia. Figure 2 presents a summary of the participants’ approaches.

Most primary care physicians described relaxing adherence to guidelines and medication regimens for other chronic conditions (e.g., hypertension, diabetes). This is because as dementia progresses, the patient’s ability to self-manage medications diminishes while the caregiver’s burden grows. Then physicians shifted their approach to prioritize patients' and caregivers' quality of life and comfort rather than the strict management of each chronic condition. Specific measures included simplifying prescriptions to reduce the number of times medication had to be taken, discontinuing medicines with a higher risk of side effects than benefits, and avoiding polypharmacy.

*“(For a patient with dementia) I think the priority of achieving the guidelines and targets for chronic diseases will be lowered; rather than aiming for the target value by taking medication twice a day, aiming for a moderate value by taking medication once a day, so that we can achieve it in a realistic way in their life with care.” (JP-R12)*

*“I might not care if their hemoglobin A1c is below 7. I really start to shift my focus to what is going to make a difference in the day-to-day quality of life for this patient and the family. And if they're on medication that might be causing side effects or is expensive, we need to re-evaluate how important it is for them.” (US-M06)*

While the approaches for multimorbidity management were similar in both countries, some different values were heard when discussing goals of care. In Michigan, “safety” was the keyword that physicians emphasized. Specifically, this meant paying attention to prevention of falls, polypharmacy, driving, housing environment (e.g., availability of handrails), food and nutrition, living alone, and guns. Michigan physicians conducted their practice in their offices, and only a few physicians were making home visits. So, the family members present at the appointment were the most important source of information about living conditions at home and making sure the patients were safe. Home visiting nurses and home care agencies had the primary roles and responsibilities in managing home care, and primary care physicians did not share such roles. Sometimes physicians were familiar with community resources assisting older people living at home (e.g., Area Office on Aging or the Council of Aging, PACE/Program of All-Inclusive Care for the Elderly, Grace program, etc.); however, they expect their care coordinators or social workers to connect their patients with such services. The cases Michigan physicians reported in the interviews were those of early and middle-stage dementia because many of older patients with dementia are institutionalized at the end of their lives and are no longer under the care of primary care physicians after institutionalization.

*It is a matter of keeping them safe and I will tell them safety is our number one priority. Prevent them from wandering out in the cold or driving if it is not safe. Also, the bulk of it takes a lot of active family involvement, oftentimes if they are still living at home. (US-U05)*

On the other hand, the keyword that Japanese physicians used as the goal of care for patients with multimorbidity who had dementia was "*Shiawase*." It means happiness, comfort, or well-being in Japanese. They observed and asked if the patients and family felt *Shiawase* and if they were living in a good relationship. This goal took precedence over the strict management of the guidelines of each chronic condition. In Japan, the government's long-term care insurance program was used to support the living environment (e.g., setting bars and arranging regular nurse and/or helper visits to home) when patients had dementia, and physicians made regular home visits to patients who could not come to the clinic. In those cases, the doctors worked as a team with visiting nurses and a care manager for each patient, sometimes holding a team conference, and they had a detailed understanding of patients’ living conditions at home and their relationships with family members and neighbors. They sometimes took care of the patients until they died at home; therefore, physicians were paying attention to whether the family was satisfied with their caregiving experience when the patient passed away.

*The goal of treatment is based on what kind of medical care is best for “Shiawase” of the family’s future in 5, 10, and 50 years. I will make sure that the patient is not in pain and reduce the day-to-day care burden of the family. On top of that, I am conscious of what the caring experience will mean to the family when this grandpa passes away. (J-R011)*

### Challenges in Japan and Michigan – The multilevel framework

Primary care physicians in Japan and Michigan reported various challenges to the implementation of the intended practice in their respective settings. We categorized their challenges into four levels, which are the environment, system, team (health care and service provider), individual (patient and family) levels, and presented them in Table 2. The following sections describe the common and unique challenges at each level in Japan and Michigan.

**Table 2:** Challenges in managing multimorbidity for patients with dementia in Japan and Michigan

Level	Common challenges	Challenges in Japan	Challenges in Michigan
Environment	-Needs for community resources, family support -Stigma to dementia	-Gap in available informal care resources in rural and urban areas	-Distance and transportation (to access health care services)
System (Medical, Insurance)	-Lack of robust management guidelines for multimorbidity patients with dementia -Short consultation time (10–15 min.) for managing multimorbidity with dementia	-System change of long-term care insurance causing a disadvantage for patients in the use of care service -Free access medical system (barrier for patient-centered, holistic management)	-Medicare covers very little
Team (Health care and service provider)	< Primary care physicians > -Complexity of adjusting medications -Concerns for polypharmacy, adverse effects -When to start, stop, or change dementia medications -Identifying different types of dementia or psychosis-associated symptoms -Managing behavioral issues	< Primary care physicians > -Approaches to the patient's living environment and local community	< Primary care physicians > -Preventing falls -The use of anticoagulant medication
	< Other professionals > -Needs for care-coordinating professionals (social workers, care managers, case managers) knowledgeable in helping patients with dementia and behavioral issues	< Other professionals > -Varied skill levels of care managers and visiting nurses -Lack of professional care resources in rural areas -Complex care management with crowded professionals in urban areas -Hospitals or facilities might not accept patients with behavioral and psychological symptoms	< Other professionals > -Need more access to specialists (Psychiatrists, Neurologists, etc.) -Need for telemedicine (Consultation with specialists) -Difficult to find a multidisciplinary team for old frail patients -Lack of information or access to home care resources (situation varies by primary care physicians' setting)
Individual (Patients and family)	-Decline of patients' self-management ability (life in general, clinical visits, medication) -Patients living alone or only with an old spouse -Patients having children or proxy in the distance (communication difficulty) -Behavioral and psychological symptoms of dementia -Patients/family not accepting or understanding dementia diagnosis -Patients or family refusing care support (stigma) -High burden on family caregivers	-Difficulty understanding the patients' will (decision-making for end-of-life care) -Dependent attitudes to doctors in decision-making	-Financial limitation of patients/family, many cannot afford good care (move into a facility, acquiring home care) -Caregivers not attending clinical visits (hard to understand patients' condition at home) -Family members not agreeing to a DNR -Cultural diversity (Language barriers, different ways of dealing with dementia)

### Environmental level

Common issues identified in Japan and Michigan were the need for community resources and caregiver support. Because family caregivers are an essential care resource for patients, physicians were always concerned about their burden.

Environmentally, Japanese physicians in rural areas reported close human relationships and informal care provided by neighbors; however, urban areas were losing such relationships. The community volunteers supporting older adults are aging and have few successors in both urban and rural areas. Each administrative district has set up a "community comprehensive support center" as a general consultation service for older adults, but there are regional differences in the proactiveness of its efforts, and most of the primary care physicians were still in the process of observing its development. Caregiving has been generally professionalized in Japan in the last 20 years, and for physicians, a challenge was how to maintain the benefits of traditional family and community ties while reducing the family's care burden via professional support.

*“On the rural island, relatives and neighbors naturally offered support. But in this urban area, there is an expectation of completing care within the family, which places a heavy burden on the family (unless using professional services).” (JP-U09)*

Michigan physicians’ major concerns in the environment were distances and transportation. Especially when patients gave up driving for safety reasons, clinical visits, access to specialists, and household chores in everyday life became challenging. Patients’ driving ability was less of a barrier to care in Japan because many participants provided home visits to patients who could no longer make clinical visits. Michigan physicians’ challenge illustrates that people’s life is more car-dependent, and because physicians are basically working in their office, not making home visits, patients’ driving ability directly affects the continuity of medical care.

*“Transportation sometimes is tough with some of them. If they can’t drive, they don’t have a family member to drive them here, so transportation to and from doctor appointments can be tough.” (US-U05)*

From the cultural standpoint, the stigma associated with dementia was mentioned by some physicians from both countries; there was a lack of understanding of dementia patients and how they need support in Michigan, and older people had a preconceived notion that it is a disability in Japan. Primary care physicians were concerned that stigma could cause hesitation by patients and families to accept the dementia diagnosis and related care support at the individual level.

### System level

There were concerns that the guidelines for various chronic conditions were not robust for patients with dementia, and disagreement among some guidelines allowed the physicians to loosen the management criteria only by the patient's age or the presence of dementia. The short consultation time was also a barrier to multimorbidity management in both locations. Many primary care physicians stated that the appointment time for one patient was 10 to 15 min, which made it challenging to assess living conditions, manage multiple chronic conditions, and consult family concerns, particularly when patients have dementia.

In Japan, universal healthcare coverage and long-term care insurance were central systems to support the lives of patients with dementia and their families. However, some Japanese physicians claimed the recent policy change toward reducing the available volume of care services for patients with low care needs. Such direction of policy change as the aging population increases can be a challenge for medication management for patients with dementia. Another topic unique in Japanese primary care was the free access to medical services, allowing patients to see any doctor without a referral causing fragmentation of care. Several participants had concerns with this system as a barrier to providing holistic care for multimorbid patients.

*“Since there are many medical practitioners in the neighborhood, the bad side of free access comes out. When I try to coordinate the whole process or what’s best for the person, I often get in trouble because the access is too good.” (JP-U13)*

In Michigan, many patients 65 years or older have Medicare, which covers annual health check-ups including cognitive functions tests and helps detect early dementia. However, in overall management, Medicare covers only limited services, so primary care physicians must arrange medical care by considering each patient's financial capability. If the patient comes to the clinic only once a year because of financial difficulties, it makes the consultation even harder to address many health issues within 15 min.

*“A reasonable proportion of my over 65 (patients) have Medicare only. They don’t have supplemental insurance. And so that’s limiting.” (US-M04)*

### Team level – Health care and service provider

#### Primary care physicians

While trying to apply relaxed adherence to chronic conditions for patients with dementia, the complexity of adjusting medication was mentioned as a physician’s challenge both in Japan and Michigan. They had concerns about polypharmacy and side effects and whether to use dementia medications or not. Identifying different types of dementia other than Alzheimer’s, or whether it is dementia or psychosis-associated symptoms were issues for which they needed advice from specialist doctors.

Participants had challenges in managing patients’ behavioral issues in both locations. In cases where patients showed symptoms of violence, agitation, or wandering, physicians sometimes spent several years dealing with the problems that arose each time by working around with family and other professionals. In some cases, it was only solved by symptoms receding naturally or patients’ sudden deaths. In some cases, primary care physicians continued to prescribe psychotropic medications initiated by psychiatrists but were reluctant to initiate these medications themselves owing to concerns about adverse effects. Physicians then intended to recommend outside services to reduce the burden on the family or to intervene in the patient's living environment during this period, although understanding the diagnosis and accepting support from care providers could sometimes be a hurdle for these patients.

Japanese physicians emphasized the importance of considering patients' living environment and interventions in the community to create supportive networks for people living with dementia. However, some participants mentioned their lack of training for managing patients' conditions from various perspectives since they learned disease-based management during their residency training.

*“When rotating specialty departments as a resident, I wasn’t taught how to manage dementia. I have come to understand later the need to incorporate nursing care in addition to medical care, including care for the family, multidisciplinary cooperation, and communication with the patient.” (JP-U04)*

Compared to Japanese physicians, Michigan physicians provided specific figures of relaxed management criteria for chronic conditions (e.g., “Hemoglobin A1c 8 percent is good enough,” “we just set the goal for 140/90 (for blood pressure).”) They were also paying close attention to the side effects of medications to prevent adverse events such as falls. Several of them mentioned that the management of anticoagulant medication was a challenge because adverse events of bleeding can be detrimental.

“There were many times where that (blood pressure) was going to overreach and get them down to 100, and then they have a fall, and I feel like I have created – I’ve done harm when I was attempting benefit. Whereas I said Okay if they can review it scientifically... just keep them over 150/90; I’m probably going to do just as much benefit in lowering their long-term risks for strokes and heart attacks.” (US-R4)

#### Other professionals

The common challenges were the need for care-coordinating professionals such as social workers, care managers, and case managers who are knowledgeable in helping patients with dementia and behavioral issues. In Japan, care managers coordinate care services using long-term care insurance. This job is a relatively new position that emerged with the long-term care insurance system since 2000 and does not require having a nursing background. Therefore, their varied skill levels, especially whether or not they had a nursing background, were concerns of primary care physicians. The mixed skill level was also reported about visiting nurses in Japan, which is another key role in caring for old patients at home. Physicians were expecting improvement and standardization in the skills of these two positions to ensure quality care.

In Michigan, on the other hand, those who had access to social workers, care coordinators, or nurses who could connect patients and families to the care resources detailed their integral role in managing patient care. However, the participants who lacked nonphysician support described a need for those positions. Sometimes, even when the positions existed, the personnel were not trained to manage patients with dementia, lessening their usefulness.

In Japan, team/organization-level challenges were different in the rural and urban areas according to the volume and density of professional care resources. In highly rural areas where the care service providers were limited, a long-term care insurance service system was sometimes not helpful. In urban areas where various care providers were active, the physicians found more challenges in communicating with the care team, which is uniquely organized for each patient with professionals from multiple organizations. Communication within the team was smoothest in a moderately remote area where multidisciplinary collaboration could be conducted within a few service organizations.

*“It takes a lot of communication effort because the care managers and home visiting nurses come from different offices, and those nurses rotate for each visit. And I think there is a greater disparity in skills (of those professions) in urban areas.” (JP-J08)*

The Japanese primary care physicians, but not the Michigan physicians, were concerned that hospitals or facilities might not accept the patients with behavioral and psychological symptoms, potentially imposing an extremely high burden on the family caregivers for a few years at home. One Japanese physician mentioned the need for more non-pharmacological care by activating community resources before the situation deteriorates.

In Michigan, many primary care physicians stressed the need for more access to specialist doctors, such as psychiatrists and neurologists, mainly to diagnose dementia and determine the different types of dementia other than Alzheimer's disease and other psychological symptoms. They were confident that family physicians could provide better care for patients with multimorbidity in a patient-centered way; however, such competency can be demonstrated under the condition that having access to specialists when needed. Because of the limited number of specialists in each area, physicians in Michigan needed telemedicine that could provide them more opportunities for consultation to specialists.

*“I think the ease of access for neuropsychologists or psychiatry is a big need right now. We’re pursuing that by trying to hire a full-time psychiatrist here, but it’s a rural area. There’s a high need and it’s just a recruiting difficulty.” (US-R11)*

Also, in Michigan, it was difficult to find a multidisciplinary care team for old and frail patients, and there was inadequate information or access to home care resources. Compared to Japanese primary care physicians providing home visits for patients who cannot visit clinics and sharing information with the multidisciplinary team, Michigan physicians seemed to recognize a clear division of roles between medical care and home care. Therefore, the lack of information sources about a patient's living condition could be challenging, especially in settings without social workers or care coordinators. Several physicians mentioned the PACE program in the university town provides overall care for frail older adults; however, the primary care physician must give up the patient for him/her to receive care from the program. The Area Agency on Aging, a publicly funded organization in each county, provided social support for older patients to maintain their independent life at home; however, the level of services could be variable depending on their funding situations.

### Individual level—patients and families

Many challenges for primary care physicians at the individual patient and family level were common in both locations. The decline in patients' self-management ability made medication management and clinical visits difficult. Patients living alone or only with an old spouse had difficulty in managing their conditions, especially in cases both patient and family had dementia. Communication with patients' children or proxies living in the distance could cause troubles such as children not understanding the seriousness of the patient's condition or disagreeing with the decisions that have already been agreed upon by other family members, the phenomenon often referred to as “daughters from California syndrome.” Patients or families who do not accept or understand the diagnosis of dementia may lose the chance to get optimal care support even if it's available. Sometimes they hesitated to seek help due to embarrassment caused by social stigma. The high burden on family caregivers was always a concern for primary care physicians.

One issue mentioned by Japanese physicians repeatedly was difficulty understanding the patients' own will for end-of-life care, while discussing most of the decision-making with the family members. In the case of physicians in Michigan, they were trying to detect dementia in the early stages and discuss advance directives when the patients were still able to express their own wills by themselves and then shifted to the communication with the family as the patient's dementia progressed. Lack of such process in early-stage dementia care in Japanese practices may have led to physicians’ regret in the decision-making at later stages. Additionally, Japanese physicians in rural areas expressed uncomfortableness with the people's attitude to rely on doctors asking them to make treatment decisions for the patient’s sake. Such value conflict with the older generations or general citizens was a challenge for younger physicians who prioritize patients' agency in treatment decision makings.

*(In advance care planning,) we explain the advantages and disadvantages of each treatment. Still, it is difficult to determine whether the person with dementia correctly understands and judges the information when voicing their wishes. (JP R07)*

In Michigan, many participants raised financial limitations of patients as barriers to care. Even when living at home alone is no longer considered safe, many people cannot afford to move into a facility or full-time home care due to financial reasons. When patients could not receive appropriate care, Japanese physicians considered it an issue of the care system or the volume of available resources in the area; however, Michigan physicians viewed it as an issue of individual responsibility and affordability at some level.

*“A lot of them are low income, or their caregivers are low income up here. So that’s sometimes difficult, just getting them in for timely appointments. I have patients who have chronic conditions who don’t come in more than once a year, because they don’t feel that they can afford it,” (US-R09)*

Michigan physicians also mentioned some difficulty in communicating with family members or caregivers not attending the clinical visits, making it hard to understand patients' condition at home and to discuss a DNR (do not resuscitate) order with family members.

Some physicians in Michigan commented on cultural diversity in language, and different ways of understanding and dealing with dementia.

*“I have a substantial number of people from the Middle East, from India, from China… and how they and their families are going to deal with dementia is substantially different from how Caucasian Americans are going to deal with dementia.” (US-M02)*

It was unique to the Michigan participants that they were facing ethnic and cultural diversity in their practice on a daily basis, while Japanese participants were mainly observing regional differences that residents in rural areas have more generous attitudes toward dementia and illness in general.

## Discussion

This study compared primary care physicians’ approaches and challenges to multimorbidity management for patients with dementia in Japan and Michigan in the United States. We found similarity in both locations that the physicians employed a relaxed adherence to guidelines for chronic conditions and prioritized the safety and comfort for the lives of patients and family. We analyzed the challenges perceived by the participants at four levels: Environmental, system, team, and individual. Several issues on system and team level challenges were identified as directly impacting primary care practices and have a potential to be improved in both locations.

First issue is the short consultation time. While our participants made efforts to adjust medical care depending on the life settings and the management capability of the patients and families, they struggled with their limited clinical consultation time in both locations when patients have multimorbidity. More time is needed with this patient group to communicate diagnoses, assess their decision-making capacity, and support their decision-making [8, 16, 19, 49, 50]. Systems that allocate more than the usual 10 to 15 min for consultation [18] or that provide a more frequent review for consulting patients with dementia [51] would be necessary considering the complexity of such care. The compensation scheme also needs to support the workload for managing multimorbidity for patients with dementia.

Second issue is the management guidelines for individual diseases lacking consideration for multimorbidity with dementia. This study identified some challenges that cannot be resolved by single disease-based guidelines, which usually offer the assumption of medication or adjustment of medication dosage [8, 12]. For example, when a patient with dementia has difficulty self-managing medication for other illnesses, physicians may need to consider alternative approaches to achieve and maintain reasonable control of those diseases. We encourage disease-based guidelines of common chronic conditions and preventive care to include information of changing priority and indicators in the case of older patients with multimorbidity with dementia. One example in Japan is the “diabetes care guide for older adults,” published in 2021, which provided adjusted goals for preventing diabetic complications according to the patient’s cognitive and physical status and medicine regimens to reduce the burden of care [52].

Third issue is the integration of health and social care for multimorbid patients with dementia [6, 7, 18, 19]. Interprofessional collaboration needs to be promoted to connect community resources and the people who need care support [8, 11, 19, 20]. Our participants’ experiences suggest that primary care physicians are more confident in providing good care for their patients with dementia when they can collaborate with skilled care managers or social workers. Especially when a patient has behavioral and psychological symptoms, non-pharmacological interventions (e.g., environmental adaptation and caregiver training) are recommended as a first-line approach [53]. In the present study, even though few participants used the term “non-pharmacological treatment,” challenges for managing behavioral issues were mentioned by many participants of both locations. Such challenge reflects the need for social workers and care coordinators who are knowledgeable for supporting dementia patients and family, and the needs for more community resources. Previous studies in the US report that clinicians consider non-pharmacological treatments to be time-consuming to explain or more burdensome to patients and caregivers, or they lack access to such services [17, 53]. This barrier may lead to inappropriate prescribing [13] including inappropriate use of antipsychotic medications for older patients with dementia [54]. To ensure primary care physicians utilize a sufficient range of care approaches for patients with dementia, they need to be supported by skilled professionals who can help coordinate non-pharmacological and social care support.

In addition to the issue described above, for physicians and families to seek appropriate support services nearby, a place or function where all the information of community care resources is consolidated within each practice area could be helpful. There were systems or organizations in Japan and Michigan that have been established to serve this purpose, such as Japan’s community-based integrated care system [40] or Area Agency on Aging in Michigan [55]. However, the cooperation with those organizations and medical institutions are still in the developmental stages [56, 57]. For caring community dwelling older adults with multimorbidity including dementia, the collaboration needs to be promoted with more access to relevant information.

 There are some potential limitations in the present study. First, regarding sampling strategy, most Japanese participants were certified family physicians by the Japan Primary Care Association, which started in 2006. Hence, these findings may not apply to more senior general practitioners in Japan who never completed systematic training in primary care experienced by younger physicians, such as in this study. The Michigan participants were limited to primary care physicians in one state. Because of the geographic vastness of the United States, the challenges, especially in environmental and team level, could vary in other states. There were no data on physicians who had refused to participate in the study. Second, for the Michigan data collection, phone interviews were used with 10 participants, and they tended to be shorter than face-to-face or video interviews, which potentially influenced the depth of data collection. Third, the four interviewers had unique backgrounds (intercultural communication in Japan, geriatric pharmacy, anthropology, and medical and public health in Michigan), which enabled multidimensional perspective in the interviews but may have influenced the interview data. Fourth, the data collection was conducted between 2015 and 2018, so that the impact of the COVID-19 (coronavirus disease 2019) pandemic had not been reflected in the results.

For future research on multimorbidity management in primary care, we suggest the use of scenario-based questions to explore the common and unique practices of decision-making approach in different social settings, considering available systems and resources, and perceived challenges in managing the multimorbidity with dementia. Such an approach can provide a basis for comparing the qualitative data and help to articulate more clearly the practical issues and socio-cultural characteristics of caring for older adults in each location. In addition, based on such research, it would be beneficial for primary care physicians to include in disease-specific guidelines the specific considerations for patients with multimorbidity, including dementia.

## Conclusions

In multimorbidity management for patients with dementia, primary care physicians in Japan and Michigan applied a relaxed adherence to the guidelines for patients’ chronic conditions. Common challenges were the suboptimal consultation time, the insufficient number or ability of care-coordinating professionals, and patients’ conditions such as difficulties with self-management, living alone, behavioral issues, and refusal of care support. Unique challenges in Japan were free-access medical systems and no advance directives or living wills discussed with the patients in end-of-life care when they still had decision-making capacity. In Michigan, physicians faced challenges of distance and lack of transportation between clinics and patients’ homes, and patients lacking the financial strength to acquire good care. To improve the quality of care for patients with multimorbidity including dementia, primary care physicians would benefit from optimal time and compensation allocated for this patient group, guidelines for chronic conditions to include information regarding changing priority for older adults with dementia, and the close collaboration of medical and social care and community resources with support of skilled care-coordinating professionals.

## Supplementary Information

**Additional file 1.**

## Data Availability

The datasets used and/or analyzed during the current study are available from the corresponding author upon reasonable request.

## Abbreviations

US: United States
LDL-C: Low density lipoprotein cholesterol
PACE: Program of All-Inclusive Care for the Elderly
DNR: Do not resuscitate
COVID-19: Coronavirus disease 2019
