# Supplementary material for: Primary care physicians' perspectives and challenges on managing multimorbidity for patients with dementia: a Japan–Michigan qualitative comparative study
Source: BMC Prim Care. 2023 Jun 27;24:132. doi: 10.1186/s12875-023-02088-4 (PMC10294331; doi:10.1186/s12875-023-02088-4)
Supplement: Supplementary file 1 — Additional file 1. [file 12875_2023_2088_MOESM1_ESM.docx]

**Supplement 1: Interview Guide**

**Key issues to address during Interview:**

- What are the attitudes and beliefs of urban primary care physicians regarding other chronic conditions in patients with dementia throughout the course of the dementing illness?
- How do urban primary care physicians make decisions about other chronic conditions in patients with dementia throughout the course of the dementing illness?
- What role, if any, does comfort with diagnosis and management of dementia play into decisions regarding management of other chronic conditions?
- For the urban primary care physician, what subspecialty resources would be most useful in the primary care of urban patients with dementia, and in what format?

**Introduction Statement:** First off, I’d like to thank you for agreeing to an interview today. My name is XXXX XXXXX, and I am coming from the University of XXXX. We feel that it is very important to speak to urban primary care physicians directly to hear about your experiences with dementia patients, so we will be conducting interviews with physicians throughout urban areas of XXXX.

During this interview today we will simply be talking about your experiences with dementia patients. I am **most interested to hear about your own personal experiences, opinions and views** on the issues we discuss, so please don’t feel shy, your views are very valuable to us, and we are **here to learn from you**. I have a list of topics I would like to discuss, but feel free to bring up any topics you feel are related to our discussion. Also, I want to let you know that **your participation is completely voluntary**, so if you want to stop at any time or don’t feel comfortable answering a question please let me know.

I would like to record our discussion so that we can review your views exactly and we don’t miss out on anything you say. Our discussion will remain **completely confidential**, only the research team will listen to the recording and the information you give will only be used for this research project to improve primary care of urban dementia patients. **Is it OK to record the discussion?**

Our interview will last about **one and a half hours**. **Are there any questions before we start?**

**Warm-Up Questions:** First, it would be nice to start with some questions about you.

1. What are some of the reasons that led you to choose a career in primary care?
2. What are some of the reasons that led you to practice in this community, rural / urban area? (probes: things enjoy/don’t enjoy)
3. How common is it to have a patient with both dementia and other chronic conditions in your practice?

**Attitudes and Beliefs About Other Conditions: Now I’d like to talk specifically about your experiences in providing primary care for patients with dementia.**

1. In your opinion, what does “primary care” of a patient with dementia look like?
2. What do you feel are your most important goals when providing primary care for a patient with dementia? (probe: why, how change, role dementia severity, role other conditions, barriers/facilitators, examples using pts how made goals)
3. What goals do you feel are less important? (probe: why, how change, role dementia severity, role other conditions, barriers/facilitators, examples using pts how made goals)

**Decision-Making & Other Conditions:** I would like you to tell me about a memorable patient of yours with dementia who also had other chronic medical conditions. I would like you to pick someone that you took care of from the beginning when suspicion emerged about memory issues, and tell me what happened over the course of providing care for them. The more details you can provide the better. Can you think of someone?

Now I’d like to draw out a timeline with the key events that you talked about.

1. Are there any other key events that occurred that we haven’t talked about already? (probes: **dx & disclosure of dementia**; the full spectrum of chronic conditions; new chronic conditions, when would it fit in the timeline)
2. What did you consider when deciding whether a particular condition should be addressed? (probe: use timeline)
3. What did you consider when managing medications? (probe: use timeline)
4. Was there anyone else involved in the decision making process when it came to addressing these other chronic conditions? (probe: family members, caretakers, specialists; how manage conflict of views)
5. Overall, how does this case compare to other patients with dementia that you have cared for? (probe: typical/not typical; other examples given: how made decisions about those conditions, medications; role of others in decision making processes)
6. Can you think of other cases, in particular, that have shaped how you address other chronic conditions in patients with dementia?
7. What impact, if any, have other chronic conditions had on end-of-life care in your patients with dementia?

**Effect of Dementia Diagnosis & Management**

1. Now I’d like to hear about your experiences with diagnosing dementia. In certain situations, some primary care providers are very comfortable in making a diagnosis of dementia, whereas some primary care providers find the diagnosis of dementia to be very hard. If 10 is the most comfortable you could possibly be with diagnosing dementia and 0 is not comfortable at all, which number would you pick?
   1. (probe: why that number? higher/lower, effect of urban)

1. Where would you place yourself on this spectrum when it comes to disclosing a diagnosis of dementia?

(probe: why that number? higher/lower, effect of urban)

1. Similar to the previous question, in certain situations, some primary care providers are very comfortable in managing patients with dementia, whereas some primary care providers find the management of dementia to be an area of great difficulty. Where would you put yourself on this spectrum and why? If 10 is the most comfortable you could possibly be with managing dementia and 0 is not comfortable at all, which number would you pick?

(probe: why that number? higher/lower, effect of urban)

1. How do you feel your comfort level with dementia relates to your ability to make decisions about other medical conditions? (probe: dx, disclosure, management)

**Sub-specialty Resources**

1. What office staff/health professionals are available to help you with your patients with dementia? (probe: most useful, staff wish available)
2. What other resources are available to help you with your patients with dementia? (probe: most useful, resources wish available)
3. If subspecialty support were available via telemedicine in your clinic to assist you with your patients with dementia, would you find this helpful? (probe: care, resources, advice; consults)
   1. IF yes: Specifically, what sort of services would find most helpful for your day to day care of these patients?
   2. If no: Could you please elaborate?
4. Would you prefer to send a patient to a consultant or, if it were possible, would you prefer to have them receive a telemedicine consult in your clinic? (probe: what see as benefits, limitations)
5. What role does home care provide for patients with dementia?

**Closing Questions:** We’re nearing towards the end of our interview, but I have a few more questions.

1. What do you feel are the three main challenges for new primary care physicians in taking care of dementia patients in rural/urban areas? What advice would you give them?
2. If you were to design an intervention to help your patients with dementia, what would you focus on? Whose input do you feel would be important?

1. Before we end, is there anything else you think I ought to know about what it is like to take care of dementia patients in rural/urban practice?

I really appreciate you taking the time to provide me with your insights about taking care of patients with dementia.
